# Opportunities for CRISPR/Cas9 Gene Editing in Retinal Regeneration Research

**DOI:** 10.3389/fcell.2017.00099

**Published:** 2017-11-23

**Authors:** Leah J. Campbell, David R. Hyde

**Affiliations:** Department of Biological Sciences, Center for Zebrafish Research and Center for Stem Cells and Regenerative Medicine, University of Notre Dame, Notre Dame, IN, United States

**Keywords:** regeneration, retina, CRISPR/Cas9, zebrafish, Müller glia, neuronal progenitor cell

## Abstract

While retinal degeneration and disease results in permanent damage and vision loss in humans, the severely damaged zebrafish retina has a high capacity to regenerate lost neurons and restore visual behaviors. Advancements in understanding the molecular and cellular basis of this regeneration response give hope that strategies and therapeutics may be developed to restore sight to blind and visually-impaired individuals. Our current understanding has been facilitated by the amenability of zebrafish to molecular tools, imaging techniques, and forward and reverse genetic approaches. Accordingly, the zebrafish research community has developed a diverse array of research tools for use in developing and adult animals, including toolkits for facilitating the generation of transgenic animals, systems for inducible, cell-specific transgene expression, and the creation of knockout alleles for nearly every protein coding gene. As CRISPR/Cas9 genome editing has begun to revolutionize molecular biology research, the zebrafish community has responded in stride by developing CRISPR/Cas9 techniques for the zebrafish as well as incorporating CRISPR/Cas9 into available toolsets. The application of CRISPR/Cas9 to retinal regeneration research will undoubtedly bring us closer to understanding the mechanisms underlying retinal repair and vision restoration in the zebrafish, as well as developing therapeutic approaches that will restore vision to blind and visually-impaired individuals. This review focuses on how CRISPR/Cas9 has been integrated into zebrafish research toolsets and how this new tool will revolutionize the field of retinal regeneration research.

## Introduction

Humans and other mammals are unable to regenerate a damaged retina, but teleost fish, such as zebrafish, possess a robust injury response where all types of retinal cells can be regenerated following loss. This is a noteworthy capacity since vision loss due to inherited or acquired retinal disease has a tremendous impact on a person's quality of life and results in substantial economic burden (Gupta et al., 2007; Wittenborn et al., 2013). Retinal damage is usually permanent and cures are nonexistent because the retina is composed of post-mitotic neurons. Over the past few decades, much work has been dedicated to developing treatments for vision loss, such as prosthetics (Barrett et al., 2014), photoreceptor transplant (Santos-Ferreira et al., 2017), and gene therapy (Farrar et al., 2014). While many of these strategies demonstrate strong potential, they are invasive and burdensome for patients and caregivers. Alternatively, regenerative medicine seeks to approach chronic disease with treatments that will stimulate repair and restore function.

Zebrafish possess a remarkably conserved eye anatomy and circuitry with most vertebrates, and like humans, the zebrafish retina is cone rich for diurnal, visually-dependent behavior (Gestri et al., 2012). Both forward and reverse genetics techniques are well established in zebrafish, making it a popular model system to study the retina. Furthermore, the recent establishment of CRISPR/Cas9 genome editing has been quickly translated to zebrafish research to streamline the efforts for introducing targeted mutations (Li et al., 2016).

Here we review CRISPR/Cas9 gene editing in relation to zebrafish retinal regeneration research. We discuss the most recent literature on retinal regeneration and the tools that have strengthened zebrafish research, including the resources available for CRISPR/Cas9 gene editing. Finally, we discuss how CRISPR/Cas9 has the potential to transform research concerning the open questions in the field of retinal regeneration.

## Current progress in retinal regeneration research

Zebrafish retinal regeneration is studied using a variety of damage models including constant intense light (Vihtelic and Hyde, 2000), selective light damage (Bernardos et al., 2007), surgical excision (Cameron, 2000), transgenic expression of the *E. coli* nitroreductase enzyme (Montgomery et al., 2010), and chemical ablation (Fimbel et al., 2007; Sherpa et al., 2008). Regardless of the damage model, Müller glia are the cells that respond to injury by dedifferentiating to a stem cell-like state. The regenerative process proceeds with asymmetric division to produce neuronal progenitor cells (NPC) and proliferation of the NPCs to replace the cells lost to damage. Comprehensive reviews are available that discuss, in depth, the current understanding of this process (Goldman, 2014; Gorsuch and Hyde, 2014; Lenkowski and Raymond, 2014; Ail and Perron, 2017). Here we review the most recent advances in the field.

In the mammalian retina, Müller glia respond to retinal damage by undergoing reactive gliosis. This response is characterized by Müller glia hypertrophy and upregulation of Glial Fibrillary Acidic Protein (GFAP) (Grosche et al., 1995; Lewis and Fisher, 2003). Although initially neuroprotective (Bringmann and Wiedemann, 2012), persistent reactive gliosis causes scarring and neuronal cell loss (Bringmann et al., 2006). Zebrafish Müller glia also respond to injury with signs of reactive gliosis such as hypertrophy and increased *gfap* expression, however this response is transient and localized to the area of damage (Thomas et al., 2016). During normal regeneration, the gliotic response transitions to Müller glia proliferation. Alternatively, inhibiting cell cycle progression in the damaged zebrafish retina increases the reactive gliosis response with upregulation of GFAP and neuroprotective genes like *fgf2* and results in the ultimate loss of photoreceptors as seen in the mammalian retina (Thomas et al., 2016). Remarkably, release of cell cycle inhibition can partially recover regeneration, further demonstrating that zebrafish Müller glia possess an enhanced capacity to respond to factors in the injured retina.

The identification of factors produced by dying neurons and the mechanisms by which zebrafish Müller glial respond have therefore been the major focus of retinal regeneration research. TNFα was the first factor identified that is produced by dying neurons and required for zebrafish Müller glia proliferation (Nelson et al., 2013). Another factor, HB-EGF, can stimulate Müller glia proliferation in some situations (Wan et al., 2012; Todd et al., 2015). Other recent studies have taken exploratory approaches to identify novel regulators of Müller glia activation following injury. Transcriptome analysis revealed previously unexamined pathways that are active in the early hours following damage including NF-κB signaling, circadian rhythm-related pathways, fatty acid metabolism, and metabolic responses (Sifuentes et al., 2016). On the protein level, cytoskeletal proteins and transporter activity appear to be upregulated in the degenerating and regenerating retinas relative to the normal retina (Eastlake et al., 2017).

Other inductive signals examined in zebrafish retinal regeneration include the core genes required to induce pluripotency in somatic cell reprogramming: *oct4, sox2, klf4, myca* and *mycb*, and *nanog* (Takahashi et al., 2007). The expression of each of these genes increases during the regenerative response (Ramachandran et al., 2010). Most recently, Sox2 expression and its functional role in Müller glia reprogramming during retinal regeneration was examined (Gorsuch et al., 2017). Through morpholino-mediated knockdown prior to light damage and forced expression in the absence of damage, it was demonstrated that Sox2 is both necessary and sufficient for Müller glia proliferation. Also necessary for Müller glia proliferation is repression of Notch signaling (Conner et al., 2014). Furthermore, it was recently suggested that Notch signaling may be regulated by Fgf8a in an age-dependent manner, such that young Müller glia respond to increased Fgf8a by repressing Notch, which allows activation and proliferation, whereas older Müller glia respond to forced Fgf8a with increased Notch signaling and neither activation nor proliferation (Wan and Goldman, 2017). This may represent an age-related preference to overcome a greater proliferative threshold in regeneration.

The strong focus on identifying the inductive signals that activate Müller glia proliferation is reasonable since a main goal for regenerative medicine is to induce Müller glia proliferation in the damaged human retina. However, for successful regeneration, Müller glia-derived NPCs must proliferate sufficiently, differentiate, and incorporate into the retina appropriately. Recent work revealed that the initial asymmetric division of Müller glia involves interkinetic nuclear migration (INM), where nuclei migrate apically to the outer nuclear layer to divide (Nagashima et al., 2013). Additionally, live-cell imaging confirmed the INM behavior of Müller glia and also revealed that most NPCs undergo apical and basal migration in phase with the cell cycle (Lahne et al., 2015). Furthermore, Rho-associated coiled-coil kinase activity, which regulates actin-myosin-mediated contraction, is required for sufficient proliferation and photoreceptor regeneration following light damage (Lahne et al., 2015). The extent to which Müller glia undergo INM may correspond to the high capacity of regeneration in the zebrafish retina.

Finally, following sufficient proliferation, successful regeneration will result only if NPCs differentiate and incorporate into the retina appropriately. While Müller glia-derived NPCs can regenerate all retinal cell types following damage, the neurons that are produced tend to be predominantly those lost to damage. Nevertheless, Müller glia-derived NPCs seem to be multipotent and regenerate neurons in excess such that all major neuronal types are produced, even when damage is focused to a particular neuronal layer of the retina (Powell et al., 2016). These newly regenerated neurons must also incorporate into the retinal circuitry. Regenerated bipolar cells appear to form most of their stereotypical connections but do not perfectly rewire with photoreceptors, suggesting that regeneration may not be able to perfectly recapitulate development (D'Orazi et al., 2016). However, the regenerated retina recovers vision-dependent behaviors (Fimbel et al., 2007; Sherpa et al., 2008), suggesting that the regenerated retina restores sufficient wiring.

## CRISPR/Cas9 gene editing in zebrafish

The great advantage of zebrafish as a model organism is that many toolsets have been developed to manipulate gene expression and protein function. In addition, the zebrafish genome is well curated and extensive databases are available that organize zebrafish strains, transgenics, and expression patterns (http://www.zfin.org/). With the development of CRISPR/Cas9 for genome editing and translation to the zebrafish system, we now have an even more powerful toolset to address open questions in retinal regeneration research.

Generating knockout mutations in zebrafish with CRISPR/Cas9 is straightforward and relatively quick. Many resources exist to assist in streamlining the process (Table 1). Two components are required: a single guide RNA (sgRNA) specific to the target sequence and the Cas9 endonuclease, which can be injected together into the zebrafish zygote. The sgRNA target sequence must be selected based on the DNA-binding requirements of Cas9, mainly a protospacer adjacent motif (PAM). The most commonly used Cas9 is from *Streptococcus pyogenes*, which requires a 5′-nGG PAM (Mojica et al., 2009). Other requirements for optimized zebrafish sgRNA sequences were incorporated into an algorithm, CRISPRscan, to select the best target for any gene of interest (Moreno-Mateos et al., 2015). Additionally, CRISPRz is a curated database of validated CRISPR targets in zebrafish (Varshney et al., 2016). Several options are available for delivery of the *cas9* transcript. Routinely, the *cas9* endonuclease is transcribed *in vitro* and injected as a mRNA. For optimal use in zebrafish, a codon-optimized *cas9* is available for expression of Cas9 protein with amino- and carboxy-terminal nuclear-localization signals (Jao et al., 2013). High efficiency activity with minimal toxicity has also been achieved with injection of an *in vitro*-assembled complex of Cas9 protein and sgRNA (Burger et al., 2016). Alternatively, transgenic zebrafish lines for ubiquitous or heat-shock inducible *cas9* expression are available, which can induce mutagenesis with sgRNA injected into the zygote or expressed via transgenic U6 promoters (Yin et al., 2015).

**Table 1:** Resources for zebrafish CRISPR/Cas9 experimental design.

**Resource**	**Description**	**Citations**
A Streamlined CRISPR Pipeline to Reliably Generate Zebrafish Frameshifting Alleles	Protocol	Talbot and Amacher, 2014
CRISPR/Cas9-mediated conversion of eGFP- into Gal4-transgenic lines in zebrafish	Protocol	Auer et al., 2014
CRISPRscan (http://www.crisprscan.org/)	sgRNA design tool	Moreno-Mateos et al., 2015
CRISPRz (https://research.nhgri.nih.gov/CRISPRz/)	Zebrafish sgRNA database	Varshney et al., 2016
Codon-optimized Cas9 (Addgene: 64237)	Plasmid	Yin et al., 2015
Vector system for tissue-specific gene disruption (Addgene: 63154, 63155, 63156, 63157)	Plasmids	Ablain et al., 2015
2C-Cas9 tool (Addgene: 74009, 74010)	Plasmids	Di Donato et al., 2016
Tg(*ubb:NLS-zCas9-NLS, myl7:EGFP*)^vu602^	Transgenic lines	Yin et al., 2015
Tg(*actb2:NLS-zCas9-NLS, cryaa:TagRFP*)^vu603^		
Tg(*fabp10a:NLS-zCas9-NLS, myl7:EGFP*)^vu604^		
Tg(*hsp70l:LOXP-mCherry-LOXP-NLS-zCas9-NLS*)^vu605^		

The Cas9/sgRNA complex induces double-stranded breaks (DSB), which typically repair via error-prone non-homologous end joining (NHEJ) to create small insertions and deletions (indels) (Lieber, 2010). This process occurs in each cell in which the Cas9-sgRNA complex is active, thereby generating a highly mosaic fish with distinct alleles in affected cells. Transheterozygotes can be analyzed for phenotype by breeding together mosaic fish injected with the same target. Alternatively, the injected mosaic fish can be out-crossed to wild type or transgenic reporter line fish to give heterozygous germline mutants, which can be bred against each other for phenotype analysis in the next generation. In some cases, the mutation efficiency may be so high that the injected mosaic fish may have mutations in both copies of the target gene and in most cells, such that the fish expresses a null-like phenotype (Jao et al., 2013).

The greatest consideration when designing and analyzing a CRISPR/Cas9 experiment should be off-target mutagenesis. Perhaps the main contributor to off-target effects is improper sgRNA target design, although the tools available to assist in design greatly reduce this risk (Talbot and Amacher, 2014; Moreno-Mateos et al., 2015; Varshney et al., 2016). Morpholino knockdown is useful as an independent and complementary technique in the analysis of CRISPR/Cas9 mutagenesis. CRISPR/Cas9 was recently used to create targeted mutations in the *neurod* gene in the zebrafish retina and to complement morpholino knockdown experiments (Taylor et al., 2015). In independent morpholino knockdown experiments, *neurod* morphants demonstrated persistent expression of the Notch receptor *notch1a*, the Notch ligands *deltaA* and *deltaD*, and the Notch targets *ascl1a* and *her4* in the central zebrafish larval retina at 48 and 70 h post fertilization. The morphants also had reduced eye size and increased proliferation zones. CRISPR/Cas9-injected embryos phenocopied the morphant phenotypes, thereby giving confidence that *neurod* was effectively targeted.

Gene targeting with CRISPR/Cas9 is not limited to a single gene. Multiple sgRNAs can be designed and coinjected into the same embryo for multigenic analysis (Jao et al., 2013; Ota et al., 2014; Yin et al., 2015). This can be useful for genetic interaction studies or as a strategy to overcome compensatory mechanisms. Multiplex gene editing with CRISPR/Cas9 may also facilitate forward and reverse genetic screening. In a pilot screen, two new genes were identified to be involved in electrical-synapse formation from a set of sgRNAs targeting 48 genes that were injected in multiplex pools of 6 or 8 sgRNAs (Shah et al., 2015). This study demonstrates potential for large-scale screening with CRISPR/Cas9.

Homology-directed repair (HDR) is another DSB repair mechanism. The molecular mechanisms that drive HDR in zebrafish are not well understood, but it requires a piece of donor DNA with homology to the area around the DSB. Therefore, targeted knock-ins can be generated at a desired locus using a donor DNA molecule that includes a sequence necessary for features such as stop codons, epitope tags, or fluorescent proteins. One report used a small oligonucleotide designed with 20 nucleotide homology arms on each side of a stop codon cassette to introduce a stop codon at the predicted DSB site at a locus where previously generated indels failed to shift the reading frame and produce a null allele (Gagnon et al., 2014). Another study used homology arms of 30–40 nucleotides to knock-in HA epitope tags directly 3′ of the start codon of two different genes with acceptable efficiency (Hruscha et al., 2013). Both frequency and precision of insertion need improvement, but recent reports show that knock-in of small insertions are successful.

Continuous effort to understand CRISPR/Cas9 function has led to the development of engineered Cas9 variants for a wide array of applications beyond inducing double-stranded breaks. For example, mutations in the endonuclease domains of the Cas9 protein render it catalytically inactive (dCas9), but expression of dCas9 with sgRNA in *E. coli* can specifically repress gene expression by blocking transcription (Qi et al., 2013). This system, called CRISPR interference (CRISPRi) has been modified for transcriptional activation or repression in eukaryotic cells by fusing dCas9 with the transcriptional activator VP64 or the repressive chromatin modifier domain, KRAB (Gilbert et al., 2013). Alternatively, the Cas9 nickase (nCas9) was engineered with a mutation to transform endonuclease activity to nickase activity where only one strand of DNA is cut (Cong et al., 2013). The nicked genomic DNA promotes repair through HDR, so recent studies have reported the use of nCas9 with two sgRNAs and homologous donor template to create specific point mutations and to correct diseasing-causing mutations (Inui et al., 2014; Kocher et al., 2017). Furthermore, the nCas9 variant has been fused to a cytidine deaminase enzyme to mediate direct conversion of C → T (or G → A) for programmable base editing (Komor et al., 2016). In zebrafish, this base editing system has been used to induce precise modifications at higher success rates than homology-directed repair (Zhang et al., 2017).

## Other zebrafish toolsets

There are several challenges that are unique to zebrafish retinal regeneration research, and certain tools have been developed to overcome those challenges. Combining the power of CRISPR/Cas9 with other tools will permit approaches to answer open questions in the field.

One big challenge for zebrafish retinal regeneration research is that the process is often studied in adult animals. While many mutant fish have been identified as having retinal defects, most are lethal within the first 2 weeks post fertilization (Brockerhoff and Fadool, 2011). Retinal regeneration experiments are, in general, designed to start with an intact, healthy retina such that gene function and experimental manipulation can be examined with respect to induced damage. In order to perform reverse genetics analyses, a method to knockdown protein function in the eye was developed using morpholino oligonucleotides (Thummel et al., 2011). Morpholinos are modified oligonucleotides that can be designed to either block translation or splicing by base-pairing to a complementary RNA. Injection and electroporation of morpholinos has facilitated the functional analysis of many genes during retinal regeneration (Thummel et al., 2010; Gramage et al., 2015; Taylor et al., 2015; Wan and Goldman, 2017), however there are some weaknesses to the technique. Mainly, electroporation has the potential to result in retinal damage outside of the experimental damage paradigm, and there is spatial limitation to the delivery of morpholino. Furthermore, there is concern that off-target effects of morpholinos may obstruct experimental analysis (Kok et al., 2015). CRISPR/Cas9 gene editing provides an independent and complementary method to confirm morpholino knockdown results.

**Figure 1:**
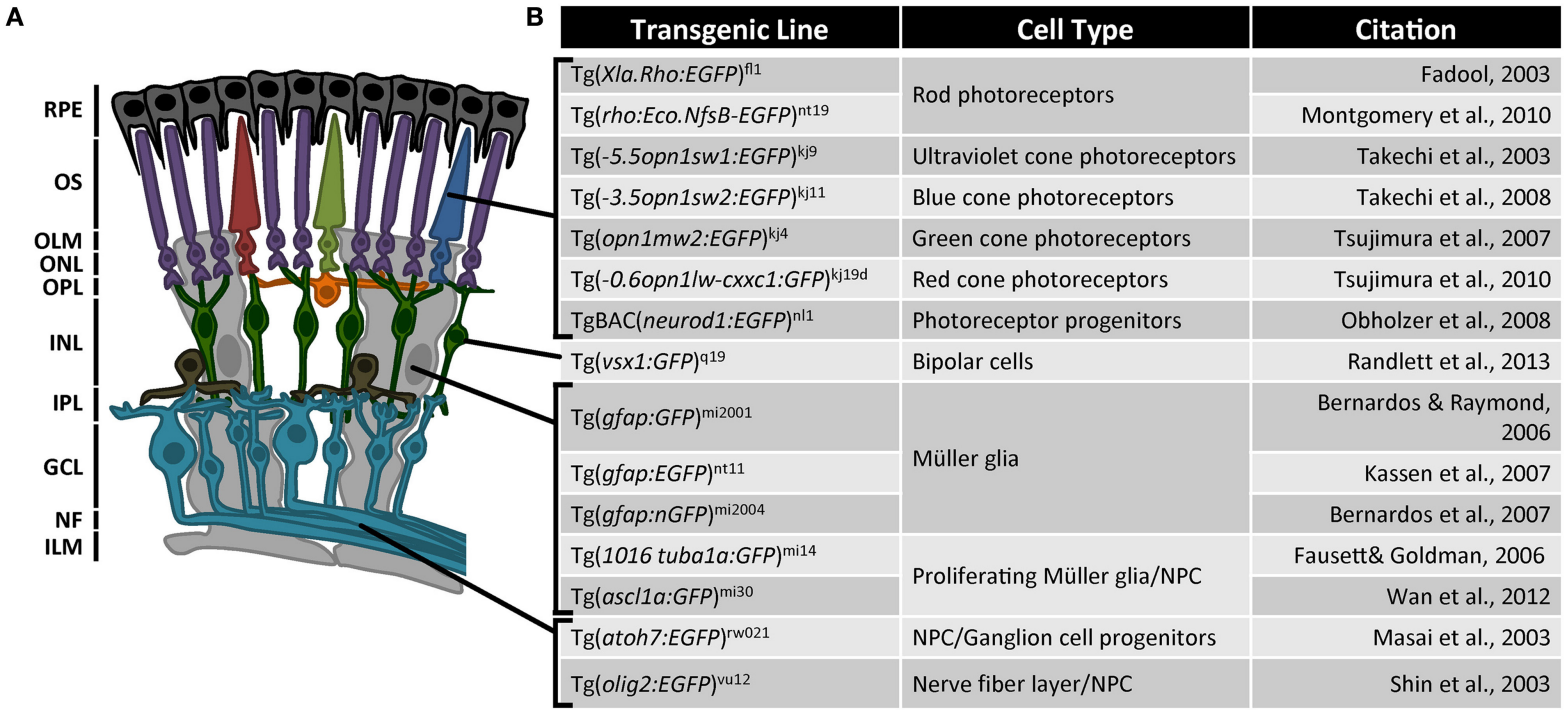
Transgenic reporter lines used in zebrafish retinal regeneration research. **(A)** The vertebrate retina is a laminated structure with different neurons located in the distinct layers. RPE, retinal pigmented epithelium; OS, outer segments; OLM, outer limiting membrane; ONL, outer nuclear layer; OPL, outer plexiform layer; INL, inner nuclear layer; IPL, inner plexiform layer; GCL, ganglion cell layer; NF, nerve fibers; ILM, inner limiting membrane. **(B)** List of transgenic reporter zebrafish lines for retinal regeneration research. Fadool (2003), Masai et al. (2003), Shin et al. (2003), Takechi et al. (2003, 2008), Bernardos and Raymond (2006), Fausett and Goldman (2006), Bernardos et al. (2007), Kassen et al. (2007), Tsujimura et al. (2007, 2010), Obholzer et al. (2008), Montgomery et al. (2010), Wan et al. (2012), Randlett et al. (2013).

Another challenge with the retina is that it is a laminated structure composed of six distinct cell types, each possessing a highly specialized function that is related to the cell's morphology and location (Figure 1A). When a gene is knocked out or knocked down in the organism or the eye, all cells lose function of that gene. Often, however, we want to know how cells relate to their environment and how genes function within time and space. Transgenic reporter lines have helped tremendously to focus on specific cell types in immunolocalization studies and for gene expression profiling by fluorescence activated cell sorting (Powell et al., 2013; Sifuentes et al., 2016). Each reporter line has utilized a specific promoter sequence for expression in a particular cell type, which provides a collection of cell-specific promoters throughout the retina (Figure 1B). Combining these cell-type specific promoters with the CRISPR/Cas9 vector system for tissue-specific gene editing will give greater precision to functional analysis in the regenerating retina (Ablain et al., 2015).

Other systems for cell-specific study include the Gal4/UAS and Tet-On gene expression systems. Both systems consist of two components: a transcriptional activator and a response element that drives expression of a reporter gene in any cell in which the transcriptional activator is active. For Gal4/UAS, the yeast transcriptional activator Gal4 binds to the *UAS* sequence, which is placed upstream of a reporter gene. A number of zebrafish Gal4 driver lines were produced through Tol2 transposon-mediated transgenesis with the Tol2kit (Kawakami et al., 2004; Kwan et al., 2007; Asakawa and Kawakami, 2008; Halpern et al., 2008). A recent protocol describes the conversion of GFP reporter lines into Gal4 lines using CRISPR/Cas9. The protocol requires a bait vector that encodes the Gal4 and includes the sgRNA target site such that the bait vector is also cut by the Cas9-sgRNA complex (Auer et al., 2014). With this technique, a GFP reporter line can be converted to a Gal4 driver line using the transgenic reporter line locus for which expression is already characterized. Furthermore, Gal4-converted transgenic lines are compatible with the 2C-Cas9 tool, a construct for clonal gene editing and tracking (Di Donato et al., 2016). The construct contains Cas9 with the self-cleaving T2A-GFP or T2A-Cre downstream of the *UAS* sequence as well as two U6 promoters to drive expression of sgRNAs.

The Tet-On system has the added benefit of being inducible in both a temporal and spatial manner. The transgene of interest is incorporated downstream of the *tetracycline response element* (*TRE*), which drives gene expression only in cells where the reverse tetracycline-controlled transcriptional transactivator (rtTA) is active and when Doxycycline is present. Transgenic zebrafish lines for inducible gene expression have been produced for rod photoreceptors [Tg(*Xla.Rho:rtTA*^*FLAG*^)^umz34^] and ultraviolet cone photoreceptors [Tg(*opn1sw1:rtTA*^*FLAG*^)^umz38^; Campbell et al., 2012; West et al., 2014]. In addition, the Tet-On components are available in Tol2kit-compatible vectors for straightforward cloning and transgenesis (Kwan et al., 2007; Campbell et al., 2012). In combination with a transgenic *TRE:Cas9*, gene editing with CRISPR/Cas9 would be inducible in a cell-type specific manner within the retina.

## Opportunities for CRISPR/Cas9 in zebrafish retinal regeneration research

Recently, much progress has been made to understand the process of retinal regeneration in zebrafish. However, we still do not yet have a sufficient understanding of the complicated molecular mechanisms that regulate the regenerative response. There are many outstanding questions that have arisen with recent results, and in time, CRISPR/Cas9 systems will play a pivotal role in addressing these questions. Targeted delivery of *ascl1a*-specific sgRNAs to the retina via injection and electroporation into one eye of Tg(*actb2:cas9;LR*)^vu603^ adult zebrafish prior to light damage demonstrated reduced number of proliferating Müller glia as compared to the corresponding control eye (Yin et al., 2015). Given its known role as an essential gene for Müller glia dedifferentiation, this result provided proof of principle that intravitreal injection and electroporation of sgRNAs can serve as a method to provide spatial and temporal control for mutagenesis in retinal regeneration studies in the adult animal.

A major area of open questions in retinal regeneration concerns the enhanced capacity of Müller glia to respond to injury. As discussed above, dying neurons produce TNFα, which is necessary and sufficient for Müller glia proliferation (Nelson et al., 2013), but TNFα has a limited capacity to induce Müller glia proliferation on its own (Conner et al., 2014). Repressing Notch signaling with a γ-secretase inhibitor along with intravitreal injection of TNFα in the undamaged retina stimulates the majority of the Müller glia to proliferate and produce NPCs that differentiate into retinal neurons (Conner et al., 2014). This suggests that not only are there inductive signals produced in the damaged retina, but that restrictive signals are also present, presumably to keep the undamaged retina in a non-proliferative state. What other factors in the zebrafish retina serve as inductive and repressive signals to permit the transient gliotic response and promote proliferation? It is most likely that a combination of factors orchestrated with particular timing creates the appropriate environment to activate Müller glia. The ability to multiplex with CRISPR/Cas9 will provide an opportunity to systematically analyze the function of one to several genes within the same retina. Further study with modified Cas9 systems, such as transcriptional repression and activation with CRISPRi, will allow for multiplex analysis of the many candidate molecules and pathways implicated in recent studies (Nelson et al., 2013; Powell et al., 2013; Sifuentes et al., 2016).

Similarly, many signaling pathways, such as Notch, β-catenin, PI3K, Jak/Stat3, and pluripotency factors, have been demonstrated to be necessary for NPC production and amplification. Recent work suggests that Müller glia do not respond equally to signals (Nelson et al., 2012; Wan and Goldman, 2017), and that they are recruited to regenerate in waves of dedifferentiation and proliferation (Gorsuch and Hyde, 2014). What are the regulatory relationships between the signaling pathways, and what defines different Müller glia cell populations? CRISPR/Cas9 gene editing combined with cell-specific and inducible expression systems will assist in answering these types of questions.

Finally, the mechanisms that direct NPCs to predominantly regenerate the specific neurons lost to damage are unclear. Recent results suggest that neurons are regenerated in excess such that neurons that were not lost to damage are produced along with those that were lost to damage (Powell et al., 2016). What signals direct the differentiation of NPCs? Does regeneration recapitulate development? Recent details and a report on the use of live imaging for retinal regeneration adds another useful tool for future studies (Lahne et al., 2017). Combining live imaging with the 2C-Cas9 tool will provide an opportunity to track cells and analyze gene function.

## Conclusions

The goal of retinal regeneration research is to translate regenerative mechanisms to humans so that a damaged retina can repair itself instead of resulting in visual impairment. Insight from zebrafish retinal regeneration research has been applied recently to the damaged mammalian retina to help reveal mechanisms that can stimulate Müller glia proliferation following damage. Ascl1 overexpression in addition to trichostatin-A (histone deacetylase inhibitor) treatment of damaged mouse retinas enabled a subset of Müller glia to proliferate and generate inner retinal neurons (Jorstad et al., 2017). Furthermore, sequencing data showed that chromatin remodeling in the responsive Müller glia was critical to express genes associated with neural development and differentiation. Continued progress with zebrafish retinal regeneration is needed to understand how to direct the complex regulatory mechanisms required for regeneration. CRISPR/Cas9 genome editing will undoubtedly play a role in the progress that will ultimately translate to visual restoration in humans who suffer visual impairment resulting from trauma or disease.

## Author contributions

LJC was the primary author of this review and DRH edited the final draft and approved the submission of the review.

### Conflict of interest statement

The authors declare that the research was conducted in the absence of any commercial or financial relationships that could be construed as a potential conflict of interest.

## References

[B1] Ablain J. Durand E. M. Yang S. Zhou Y. Zon L. I. (2015). A CRISPR/Cas9 vector system for tissue-specific gene disruption in zebrafish. Dev. Cell 32, 756–764. 10.1016/j.devcel.2015.01.032 25752963 PMC4379706

[B2] Ail D. Perron M. (2017). Retinal degeneration and regeneration-lessons from fishes and Amphibians. Curr. Pathobiol. Rep. 5, 67–78. 10.1007/s40139-017-0127-9 28255526 PMC5309292

[B3] Asakawa K. Kawakami K. (2008). Targeted gene expression by the Gal4-UAS system in zebrafish. Dev. Growth Differ. 50, 391–399. 10.1111/j.1440-169X.2008.01044.x 18482403

[B4] Auer T. O. Duroure K. Concordet J. P. Del Bene F. (2014). CRISPR/Cas9-mediated conversion of eGFP- into Gal4-transgenic lines in zebrafish. Nat. Protoc. 9, 2823–2840. 10.1038/nprot.2014.187 25393779

[B5] Barrett J. M. Berlinguer-Palmini R. Degenaar P. (2014). Optogenetic approaches to retinal prosthesis. Vis. Neurosci. 31, 345–354. 10.1017/S0952523814000212 25100257 PMC4161214

[B6] Bernardos R. L. Barthel L. K. Meyers J. R. Raymond P. A. (2007). Late-stage neuronal progenitors in the retina are radial Müller glia that function as retinal stem cells. J. Neurosci. 27, 7028–7040. 10.1523/JNEUROSCI.1624-07.2007 17596452 PMC6672216

[B7] Bernardos R. L. Raymond P. A. (2006). GFAP transgenic zebrafish. Gene Expr. Patterns 6, 1007–1013. 10.1016/j.modgep.2006.04.006 16765104

[B8] Bringmann A. Pannicke T. Grosche J. Francke M. Wiedemann P. Skatchkov S. N.. (2006). Müller cells in the healthy and diseased retina. Prog. Retin. Eye Res. 25, 397–424. 10.1016/j.preteyeres.2006.05.003 16839797

[B9] Bringmann A. Wiedemann P. (2012). Müller glial cells in retinal disease. Ophthalmologica 227, 1–19. 10.1159/000328979 21921569

[B10] Brockerhoff S. E. Fadool J. M. (2011). Genetics of photoreceptor degeneration and regeneration in zebrafish. Cel. Mol. Life Sci. 68, 651–659. 10.1007/s00018-010-0563-8 20972813 PMC3029675

[B11] Burger A. Lindsay H. Felker A. Hess C. Anders C. Chiavacci E.. (2016). Maximizing mutagenesis with solubilized CRISPR-Cas9 ribonucleoprotein complexes. Development 143, 2025–2037. 10.1242/dev.134809 27130213

[B12] Cameron D. A. (2000). Cellular proliferation and neurogenesis in the injured retina of adult zebrafish. Vis. Neurosci. 17, 789–797. 10.1017/S0952523800175121 11153658

[B13] Campbell L. J. Willoughby J. J. Jensen A. M. (2012). Two types of Tet-On transgenic lines for doxycycline-inducible gene expression in zebrafish rod photoreceptors and a gateway-based tet-on toolkit. PLoS ONE 7:e51270. 10.1371/journal.pone.0051270 23251476 PMC3520995

[B14] Cong L. Ran F. A. Cox D. Lin S. Barretto R. Habib N.. (2013). Multiplex genome engineering using CRISPR/Cas systems. Science 339, 819–823. 10.1126/science.1231143 23287718 PMC3795411

[B15] Conner C. Ackerman K. M. Lahne M. Hobgood J. S. Hyde D. R. (2014). Repressing notch signaling and expressing TNFα are sufficient to mimic retinal regeneration by inducing Müller glial proliferation to generate committed progenitor cells. J. Neurosci. 34, 14403–14419. 10.1523/JNEUROSCI.0498-14.2014 25339752 PMC4205560

[B16] Di Donato V. De Santis F. Auer T. O. Testa N. Sanchez-Iranzo H. Mercader N.. (2016). 2C-Cas9: a versatile tool for clonal analysis of gene function. Genome Res. 26, 681–692. 10.1101/gr.196170.115 26957310 PMC4864464

[B17] D'Orazi F. D. Zhao X. F. Wong R. O. Yoshimatsu T. (2016). Mismatch of synaptic patterns between neurons produced in regeneration and during development of the vertebrate retina. Curr. Biol. 26, 2268–2279. 10.1016/j.cub.2016.06.063 27524481 PMC5534240

[B18] Eastlake K. Heywood W. E. Tracey-White D. Aquino E. Bliss E. Vasta G. R.. (2017). Comparison of proteomic profiles in the zebrafish retina during experimental degeneration and regeneration. Sci. Rep. 7:44601. 10.1038/srep44601 28300160 PMC5353638

[B19] Fadool J. M. (2003). Development of a rod photoreceptor mosaic revealed in transgenic zebrafish. Dev. Biol. 258, 277–290. 10.1016/S0012-1606(03)00125-8 12798288

[B20] Farrar G. J. Millington-Ward S. Chadderton N. Mansergh F. C. Palfi A. (2014). Gene therapies for inherited retinal disorders. Vis. Neurosci. 31, 289–307. 10.1017/S0952523814000133 24949856

[B21] Fausett B. V. Goldman D. (2006). A Role for α1 tubulin-expressing Müller glia in regeneration of the injured zebrafish retina. J. Neurosci. 26, 6303–6313. 10.1523/JNEUROSCI.0332-06.2006 16763038 PMC6675181

[B22] Fimbel S. M. Montgomery J. E. Burket C. T. Hyde D. R. (2007). Regeneration of inner retinal neurons after intravitreal injection of ouabain in zebrafish. J. Neurosci. 27, 1712–1724. 10.1523/JNEUROSCI.5317-06.2007 17301179 PMC6673754

[B23] Gagnon J. A. Valen E. Thyme S. B. Huang P. Ahkmetova L. Pauli A.. (2014). Efficient mutagenesis by Cas9 protein-mediated oligonucleotide insertion and large-scale assessment of single-guide RNAs. PLoS ONE 9:e98186. 10.1371/journal.pone.0098186 24873830 PMC4038517

[B24] Gestri G. Link B. A. Neuhauss S. C. (2012). The visual system of zebrafish and its use to model human ocular diseases. Dev. Neurobiol. 72, 302–327. 10.1002/dneu.20919 21595048 PMC3202066

[B25] Gilbert L. A. Larson M. H. Morsut L. Liu Z. Brar G. A. Torres S. E.. (2013). CRISPR-mediated modular RNA-guided regulation of transcription in eukaryotes. Cell 154, 442–451. 10.1016/j.cell.2013.06.044 23849981 PMC3770145

[B26] Goldman D. (2014). Müller glial cell reprogramming and retina regeneration. Nat. Rev. Neurosci. 15, 431–442. 10.1038/nrn3723 24894585 PMC4249724

[B27] Gorsuch R. A. Hyde D. R. (2014). Regulation of Müller glial dependent neuronal regeneration in the damaged adult zebrafish retina. Exp. Eye Res. 123, 131–140. 10.1016/j.exer.2013.07.012 23880528 PMC3877724

[B28] Gorsuch R. A. Lahne M. Yarka C. E. Petravick M. E. Li J. Hyde D. R. (2017). Sox2 regulates Müller glia reprogramming and proliferation in the regenerating zebrafish retina via Lin28 and Ascl1a. Exp. Eye Res. 161, 174–192. 10.1016/j.exer.2017.05.012 28577895 PMC5554723

[B29] Gramage E. D'Cruz T. Taylor S. Thummel R. Hitchcock P. F. (2015). Midkine-a protein localization in the developing and adult retina of the zebrafish and its function during photoreceptor regeneration. PLoS ONE 10:e0121789. 10.1371/journal.pone.0121789 25803551 PMC4372396

[B30] Grosche J. Härtig W. Reichenbach A. (1995). Expression of glial fibrillary acidic protein (GFAP), glutamine synthetase (GS), and Bcl-2 protooncogene protein by Müller (glial) cells in retinal light damage of rats. Neurosci. Lett. 185, 119–122. 10.1016/0304-3940(94)11239-F 7746501

[B31] Gupta O. P. Brown G. C. Brown M. M. (2007). Age-related macular degeneration: the costs to society and the patient. Curr. Opin. Ophthalmol. 18, 201–205. 10.1097/ICU.0b013e32810c8df4 17435426

[B32] Halpern M. E. Rhee J. Goll M. G. Akitake C. M. Parsons M. Leach S. D. (2008). Gal4/UAS transgenic tools and their application to zebrafish. Zebrafish 5, 97–110. 10.1089/zeb.2008.0530 18554173 PMC6469517

[B33] Hruscha A. Krawitz P. Rechenberg A. Heinrich V. Hecht J. Haass C.. (2013). Efficient CRISPR/Cas9 genome editing with low off-target effects in zebrafish. Development 140, 4982–4987. 10.1242/dev.099085 24257628

[B34] Inui M. Miyado M. Igarashi M. Tamano M. Kubo A. Yamashita S.. (2014). Rapid generation of mouse models with defined point mutations by the CRISPR/Cas9 system. Sci. Rep. 4:5396. 10.1038/srep05396 24953798 PMC4066261

[B35] Jao L. E. Wente S. R. Chen W. (2013). Efficient multiplex biallelic zebrafish genome editing using a CRISPR nuclease system. Proc. Natl. Acad. Sci. U.S.A. 110, 13904–13909. 10.1073/pnas.1308335110 23918387 PMC3752207

[B36] Jorstad N. L. Wilken M. S. Grimes W. N. Wohl S. G. VandenBosch L. S. Yoshimatsu T.. (2017). Stimulation of functional neuronal regeneration from Müller glia in adult mice. Nature 548, 103–107. 10.1038/nature23283 28746305 PMC5991837

[B37] Kassen S. C. Ramanan V. Montgomery J. E. Burket T. C. Liu C. G. Vihtelic T. S.. (2007). Time course analysis of gene expression during light-induced photoreceptor cell death and regeneration in albino zebrafish. Dev. Neurobiol. 67, 1009–1031. 10.1002/dneu.20362 17565703

[B38] Kawakami K. Takeda H. Kawakami N. Kobayashi M. Matsuda N. Mishina M. (2004). A transposon-mediated gene trap approach identifies developmentally regulated genes in zebrafish. Dev. Cell 7, 133–144. 10.1016/j.devcel.2004.06.005 15239961

[B39] Kocher T. Peking P. Klausegger A. Murauer E. M. Hofbauer J. P. Wally V.. (2017). Cut and paste: efficient homology-directed repair of a dominant negative KRT14 mutation via CRISPR/Cas9 nickases. Mol. Ther. 25, 2585–2598. 10.1016/j.ymthe.2017.08.015 28888469 PMC5675592

[B40] Kok F. O. Shin M. Ni C.-W. Gupta A. Grosse A. S. van Impel A.. (2015). Reverse genetic screening reveals poor correlation between morpholino-induced and mutant phenotypes in zebrafish. Dev. Cell 32, 97–108. 10.1016/j.devcel.2014.11.018 25533206 PMC4487878

[B41] Komor A. C. Kim Y. B. Packer M. S. Zuris J. A. Liu D. R. (2016). Programmable editing of a target base in genomic DNA without double-stranded DNA cleavage. Nature 533, 420–424. 10.1038/nature17946 27096365 PMC4873371

[B42] Kwan K. M. Fujimoto E. Grabher C. Mangum B. D. Hardy M. E. Campbell D. S.. (2007). The Tol2kit: a multisite Gateway-based construction kit for Tol2 transposon transgenesis constructs. Dev. Dyn. 236, 3088–3099. 10.1002/dvdy.21343 17937395

[B43] Lahne M. Gorsuch R. A. Nelson C. M. Hyde D. R. (2017). Culture of adult transgenic zebrafish retinal explants for live-cell imaging by multiphoton microscopy. J. Vis. Exp. 120:e55335 10.3791/55335 PMC5409321 28287581

[B44] Lahne M. Li J. Marton R. M. Hyde D. R. (2015). Actin-Cytoskeleton- and rock-mediated INM are required for photoreceptor regeneration in the adult zebrafish retina. J. Neurosci. 35, 15612–15634. 10.1523/JNEUROSCI.5005-14.2015 26609156 PMC4659825

[B45] Lenkowski J. R. Raymond P. A. (2014). Müller glia: stem cells for generation and regeneration of retinal neurons in teleost fish. Prog. Retin. Eye Res. 40, 94–123. 10.1016/j.preteyeres.2013.12.007 24412518 PMC3999222

[B46] Lewis G. P. Fisher S. K. (2003). Up-regulation of glial fibrillary acidic protein in response to retinal injury: its potential role in glial remodeling and a comparison to vimentin expression. Int. Rev. Cytol. 230, 263–290. 10.1016/S0074-7696(03)30005-1 14692684

[B47] Li M. Zhao L. Page-McCaw P. S. Chen W. (2016). Zebrafish Genome engineering using the CRISPR–Cas9 system. Trends Genet. 32, 815–827. 10.1016/j.tig.2016.10.005 27836208 PMC5127170

[B48] Lieber M. R. (2010). The mechanism of double-strand DNA break repair by the nonhomologous DNA end-joining pathway. Annu. Rev. Biochem. 79, 181–211. 10.1146/annurev.biochem.052308.093131 20192759 PMC3079308

[B49] Masai I. Lele Z. Yamaguchi M. Komori A. Nakata A. Nishiwaki Y.. (2003). N-cadherin mediates retinal lamination, maintenance of forebrain compartments and patterning of retinal neurites. Development 130, 2479–2494. 10.1242/dev.00465 12702661

[B50] Mojica F. J. M. Díez-Villaseñor C. García-Martínez J. Almendros C. (2009). Short motif sequences determine the targets of the prokaryotic CRISPR defence system. Microbiology 155, 733–740. 10.1099/mic.0.023960-0 19246744

[B51] Montgomery J. E. Parsons M. J. Hyde D. R. (2010). A novel model of retinal ablation demonstrates that the extent of rod cell death regulates the origin of the regenerated zebrafish rod photoreceptors. J. Comp. Neurol. 518, 800–814. 10.1002/cne.22243 20058308 PMC3656417

[B52] Moreno-Mateos M. A. Vejnar C. E. Beaudoin J.-D. Fernandez J. P. Mis E. K. Khokha M. K.. (2015). CRISPRscan: designing highly efficient sgRNAs for CRISPR-Cas9 targeting *in vivo*. Nat. Methods 12, 982–988. 10.1038/nmeth.3543 26322839 PMC4589495

[B53] Nagashima M. Barthel L. K. Raymond P. A. (2013). A self-renewing division of zebrafish Müller glial cells generates neuronal progenitors that require N-cadherin to regenerate retinal neurons. Development 140, 4510–4521. 10.1242/dev.090738 24154521 PMC3817940

[B54] Nelson C. M. Ackerman K. M. O'Hayer P. Bailey T. J. Gorsuch R. A. Hyde D. R. (2013). tumor necrosis factor-alpha is produced by dying retinal neurons and is required for Müller glia proliferation during zebrafish retinal regeneration. J. Neurosci. 33, 6524–6539. 10.1523/JNEUROSCI.3838-12.2013 23575850 PMC3740543

[B55] Nelson C. M. Gorsuch R. A. Bailey T. J. Ackerman K. M. Kassen S. C. Hyde D. R. (2012). Stat3 defines three populations of müller glia and is required for initiating maximal müller glia proliferation in the regenerating zebrafish retina. J. Comp. Neurol. 520, 4294–4311. 10.1002/cne.23213 22886421 PMC3478445

[B56] Obholzer N. Wolfson S. Trapani J. G. Mo W. Nechiporuk A. Busch-Nentwich E.. (2008). Vesicular glutamate transporter 3 is required for synaptic transmission in zebrafish hair cells. J. Neurosci. 28, 2110–2118. 10.1523/JNEUROSCI.5230-07.2008 18305245 PMC6671858

[B57] Ota S. Hisano Y. Ikawa Y. Kawahara A. (2014). Multiple genome modifications by the CRISPR/Cas9 system in zebrafish. Genes Cells 19, 555–564. 10.1111/gtc.12154 24848337

[B58] Powell C. Cornblath E. Elsaeidi F. Wan J. Goldman D. (2016). Zebrafish Müller glia-derived progenitors are multipotent, exhibit proliferative biases and regenerate excess neurons. Sci. Rep. 6:24851. 10.1038/srep24851 27094545 PMC4837407

[B59] Powell C. Grant A. R. Cornblath E. Goldman D. (2013). Analysis of DNA methylation reveals a partial reprogramming of the Müller glia genome during retina regeneration. Proc. Natl. Acad. Sci. U.S.A. 110, 19814–19819. 10.1073/pnas.1312009110 24248357 PMC3856824

[B60] Qi L. S. Larson M. H. Gilbert L. A. Doudna J. A. Weissman J. S. Arkin A. P.. (2013). Repurposing CRISPR as an RNA-guided platform for sequence-specific control of gene expression. Cell 152, 1173–1183. 10.1016/j.cell.2013.02.022 23452860 PMC3664290

[B61] Ramachandran R. Fausett B. V. Goldman D. (2010). Ascl1a regulates Müller glia dedifferentiation and retinal regeneration through a Lin-28-dependent, let-7 microRNA signalling pathway. Nat. Cell Biol. 12, 1101–1107. 10.1038/ncb2115 20935637 PMC2972404

[B62] Randlett O. MacDonald R. B. Yoshimatsu T. Almeida A. D. Suzuki S. C. Wong R. O.. (2013). Cellular requirements for building a retinal neuropil. Cell Rep. 3, 282–290. 10.1016/j.celrep.2013.01.020 23416047 PMC3607253

[B63] Santos-Ferreira T. F. Borsch O. Ader M. (2017). Rebuilding the missing part—a review on photoreceptor transplantation. Front. Syst. Neurosci. 10:105. 10.3389/fnsys.2016.00105 28105007 PMC5214672

[B64] Shah A. N. Davey C. F. Whitebirch A. C. Miller A. C. Moens C. B. (2015). Rapid reverse genetic screening using CRISPR in zebrafish. Nat. Methods 12, 535–540. 10.1038/nmeth.3360 25867848 PMC4667794

[B65] Sherpa T. Fimbel S. M. Mallory D. E. Maaswinkel H. Spritzer S. D. Sand J. A.. (2008). Ganglion cell regeneration following whole-retina destruction in zebrafish. Dev. Neurobiol. 68, 166–181. 10.1002/dneu.20568 18000816 PMC2581885

[B66] Shin J. Park H.-C. Topczewska J. M. Mawdsley D. J. Appel B. (2003). Neural cell fate analysis in zebrafish using olig2 BAC transgenics. Methods Cell Sci. 25, 7–14. 10.1023/B:MICS.0000006847.09037.3a 14739582

[B67] Sifuentes C. J. Kim J. W. Swaroop A. Raymond P. A. (2016). Rapid, dynamic activation of müller glial stem cell responses in zebrafish. Invest. Ophthalmol. Vis. Sci. 57, 5148–5160. 10.1167/iovs.16-19973 27699411 PMC5054728

[B68] Takahashi K. Tanabe K. Ohnuki M. Narita M. Ichisaka T. Tomoda K.. (2007). Induction of pluripotent stem cells from adult human fibroblasts by defined factors. Cell 131, 861–872. 10.1016/j.cell.2007.11.019 18035408

[B69] Takechi M. Hamaoka T. Kawamura S. (2003). Fluorescence visualization of ultraviolet-sensitive cone photoreceptor development in living zebrafish. FEBS Lett. 553, 90–94. 10.1016/S0014-5793(03)00977-3 14550552

[B70] Takechi M. Seno S. Kawamura S. (2008). Identification of cis-acting elements repressing blue opsin expression in zebrafish UV cones and pineal cells. J. Biol. Chem. 283, 31625–31632. 10.1074/jbc.M806226200 18796431

[B71] Talbot J. C. Amacher S. L. (2014). A Streamlined CRISPR pipeline to reliably generate zebrafish frameshifting alleles. Zebrafish 11, 583–585. 10.1089/zeb.2014.1047 25470533 PMC4248249

[B72] Taylor S. M. Alvarez-Delfin K. Saade C. J. Thomas J. L. Thummel R. Fadool J. M. (2015). The bHLH transcription factor neurod governs photoreceptor genesis and regeneration through delta-notch signalingneurod regulation of photoreceptor genesis. Invest. Ophthalmol. Vis. Sci. 56, 7496–7515. 10.1167/iovs.15-17616 26580854 PMC4654396

[B73] Thomas J. L. Ranski A. H. Morgan G. W. Thummel R. (2016). Reactive gliosis in the adult zebrafish retina. Exp. Eye Res. 143, 98–109. 10.1016/j.exer.2015.09.017 26492821

[B74] Thummel R. Bailey T. J. Hyde D. R. (2011). *In vivo* electroporation of morpholinos into the adult zebrafish retina. J. Vis. Exp. e3603 10.3791/3603 22231802 PMC3369653

[B75] Thummel R. Enright J. M. Kassen S. C. Montgomery J. E. Bailey T. J. Hyde D. R. (2010). Pax6a and Pax6b are required at different points in neuronal progenitor cell proliferation during zebrafish photoreceptor regeneration. Exp. Eye Res. 90, 572–582. 10.1016/j.exer.2010.02.001 20152834 PMC2856924

[B76] Todd L. Volkov L. I. Zelinka C. Squires N. Fischer A. J. (2015). Heparin-binding EGF-like growth factor (HB-EGF) stimulates the proliferation of Müller glia-derived progenitor cells in avian and murine retinas. Mol. Cell. Neurosci. 69, 54–64. 10.1016/j.mcn.2015.10.004 26500021 PMC4658256

[B77] Tsujimura T. Chinen A. Kawamura S. (2007). Identification of a locus control region for quadruplicated green-sensitive opsin genes in zebrafish. Proc. Natl. Acad. Sci. U.S.A. 104, 12813–12818. 10.1073/pnas.0704061104 17646658 PMC1937549

[B78] Tsujimura T. Hosoya T. Kawamura S. (2010). A Single Enhancer regulating the differential expression of duplicated red-sensitive opsin genes in zebrafish. PLoS Genet. 6:e1001245. 10.1371/journal.pgen.1001245 21187910 PMC3002997

[B79] Varshney G. K. Zhang S. Pei W. Adomako-Ankomah A. Fohtung J. Schaffer K.. (2016). CRISPRz: a database of zebrafish validated sgRNAs. Nucleic Acids Res. 44, D822–D826. 10.1093/nar/gkv998 26438539 PMC4702947

[B80] Vihtelic T. S. Hyde D. R. (2000). Light-induced rod and cone cell death and regeneration in the adult albino zebrafish (*Danio rerio*) retina. J. Neurobiol. 44, 289–307. 10.1002/1097-4695(20000905)44:3<289::AID-NEU1>3.0.CO;2-H 10942883

[B81] Wan J. Goldman D. (2017). Opposing actions of Fgf8a on notch signaling distinguish two Müller glial cell populations that contribute to retina growth and regeneration. Cell Rep. 19, 849–862. 10.1016/j.celrep.2017.04.009 28445734 PMC5467687

[B82] Wan J. Ramachandran R. Goldman D. (2012). HB-EGF Is Necessary and sufficient for Müller glia dedifferentiation and retina regeneration. Dev. Cell 22, 334–347. 10.1016/j.devcel.2011.11.020 22340497 PMC3285435

[B83] West M. C. Campbell L. J. Willoughby J. J. Jensen A. M. (2014). Two types of transgenic lines for doxycycline-inducible, cell-specific gene expression in zebrafish ultraviolet cone photoreceptors. Gene Expr. Patterns 14, 96–104. 10.1016/j.gep.2014.01.002 24462722 PMC3976903

[B84] Wittenborn J. S. Zhang X. Feagan C. W. Crouse W. L. Shrestha S. Kemper A. R.. (2013). The economic burden of vision loss and eye disorders among the United States population younger than 40 years. Ophthalmology 120, 1728–1735. 10.1016/j.ophtha.2013.01.068 23631946 PMC5304763

[B85] Yin L. Maddison L. A. Li M. Kara N. LaFave M. C. Varshney G. K.. (2015). Multiplex conditional mutagenesis using transgenic expression of Cas9 and sgRNAs. Genetics 200, 431–441. 10.1534/genetics.115.176917 25855067 PMC4492370

[B86] Zhang Y. Qin W. Lu X. Xu J. Huang H. Bai H.. (2017). Programmable base editing of zebrafish genome using a modified CRISPR-Cas9 system. Nat. Commun. 8:118. 10.1038/s41467-017-00175-6 28740134 PMC5524635

